# Anti-Aging Effect of *Momordica charantia* L. on d-Galactose-Induced Subacute Aging in Mice by Activating PI3K/AKT Signaling Pathway

**DOI:** 10.3390/molecules27144502

**Published:** 2022-07-14

**Authors:** Dongxue Wang, Enhui Wang, Ying Li, Yaran Teng, Hui Li, Lili Jiao, Wei Wu

**Affiliations:** Jilin Ginseng Academy, Changchun University of Chinese Medicine, Changchun 130117, China; wangdongxue021@163.com (D.W.); m15144146451@163.com (E.W.); ying060111@163.com (Y.L.); tyr313006979@163.com (Y.T.); lihuiterrisa@163.com (H.L.); jiaoaj@hotmail.com (L.J.)

**Keywords:** *Momordica charantia* L., UHPLC-MS, aging, antioxidant, PI3K/AKT signaling pathway

## Abstract

Anti-aging is a challenging and necessary research topic. *Momordica charantia* L. is a common edible medicinal plant that has various pharmacological activities and is often employed in daily health care. However, its anti-aging effect on mice and the underlying mechanism thereof remain unclear. Our current study mainly focused on the effect of *Momordica charantia* L. on d-galactose-induced subacute aging in mice and explored the underlying mechanism. UHPLC-Q-Exactive Orbitrap MS was applied to qualitatively analyze the chemical components of *Momordica charantia* L. ethanol extract (MCE). A subacute aging mice model induced by d-galactose (d-gal) was established to investigate the anti-aging effect and potential mechanism of MCE. The learning and memory ability of aging mice was evaluated using behavioral tests. The biochemical parameters, including antioxidant enzyme activity and the accumulation of lipid peroxides in serum, were measured to explore the effect of MCE on the redox imbalance caused by aging. Pathological changes in the hippocampus were observed using hematoxylin and eosin (H&E) staining, and the levels of aging-related proteins in the PI3K/AKT signaling pathway were assessed using Western blotting. The experimental results demonstrated that a total of 14 triterpenoids were simultaneously identified in MCE. The behavioral assessments results showed that MCE can improve the learning and memory ability of subacute mice. The biochemical parameters determination results showed that MCE can improve the activity of antioxidant enzymes and decrease the accumulation of lipid peroxides in aging mice significantly. Furthermore, aging and injury in the hippocampus were ameliorated. Mechanistically, the results showed a significant upregulation in the protein expression of P-PI3K/PI3K and P-AKT/AKT (*p* < 0.01), as well as a significant reduction in cleaved caspase-3/caspase-3, Bax and P-mTOR/mTOR (*p* < 0.01). Our results confirm that MCE could restore the antioxidant status and improve cognitive impairment in aging mice, inhibit d-gal-induced apoptosis by regulating the PI3K/AKT signaling pathway, and rescue the impaired autophagy caused by mTOR overexpression, thereby exerting an anti-aging effect.

## 1. Introduction

Aging, from the beginning of life, is a complex, irreversible, multifactorial and inevitable physiological activity that everyone must experience. From a biological point of view, aging is the progressive degradation of physical compositions and tissue structures accompanied by a gradually loss of physiological functions. From a pathological viewpoint, aging is the accumulation of stress, injury, strain, immune decline, metabolic disorders and so on. It is continuous, causing a slowdown in metabolic rate, a decrease in body and organ function, and an increase in the prevalence of related diseases, including cardiovascular and cerebrovascular diseases, tumors and neurodegenerative diseases, making it considerably challenging to stay healthy [1]. Delaying aging has always been an appealing study topic. A variety of drugs have been confirmed to have a certain anti-aging effect, including metformin, rapamycin and procaine, etc. [2,3,4]. However, their long-term use may be restricted by side effects. Medicinal herbs and functional edible herbs have gradually attracted more attention in the field of anti-aging due to their multiple compounds and targets [5].

*Momordica charantia* L., commonly known as bitter melon, balsam pear and bitter gourd, is annual herbaceous climbing plant belonging to the family Cucurbitaceae. It originated in Asia but is more common in India and the south of China. It is widely planted in tropical and subtropical regions because of its high nutritional value and various pharmacological effects [6]. It can be cooked directly as daily food or processed into tea or wine for drinking. In addition, it is also used as a medicine in many areas [7]. Bioactive compounds, including terpenoids, saponins, flavonoids, sterols, glycosides, phenols, etc., have been isolated from the seeds, fruits and leaves of *Momordica charantia* L. [8], and these contribute to the various antidiabetic, anticancer, antiviral, antioxidant, antimalarial, anti-inflammatory, neuroprotective and immunomodulatory activities [9,10,11,12,13,14,15].

Many discoveries and applications of medicinal herbs in the anti-aging field were based on aging theories such as the free radical theory, the cross-linking theory, the mitochondrial DNA damage theory and immunosenescence theory, etc. [16,17,18,19]. At present, studies have shown that saponins of *Momordica charantia* L. could significantly prolong the lifespan of *Caenorhabditis elegans* and yeast by alleviating the state of oxidative stress [20,21]. However, excessive free radicals cause not only oxidative stress and redox imbalance but also apoptosis [22,23]. Numerous reports have suggested that aging is closely related to apoptosis and autophagy [24,25]. Based on this consideration, the PI3K/AKT signaling pathway, which has a regulatory effect on survival, apoptosis and autophagy, was selected for our experiment.

In this study, firstly, ultra-high-performance liquid chromatography coupled with quadrupole exactive orbitrap mass spectrometry (UHPLC-Q-Exative Orbitrap MS) was used for a qualitative analysis of the chemical components in *Momordica charantia* L. ethanol extract (MCE). The effect of MCE on subacute aging mice was then studied from the following aspects: behavioral assessments, antioxidant status and a histopathological observation of the hippocampus and the anti-aging mechanism, in a bid to verify the anti-aging effect and provide the potential mechanisms of *Momordica charantia* L.

## 2. Results

### 2.1. UHPLC-MS Analysis

**Figure 1:**
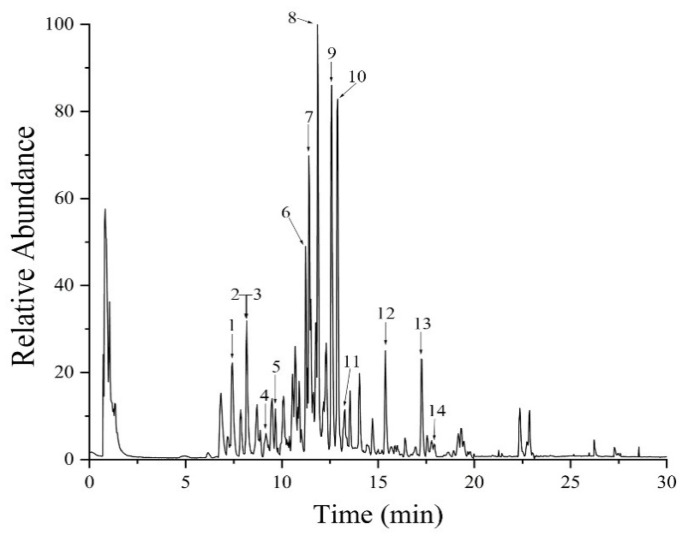
UHPLC-MS TIC of MCE. (Numbers 1–14 represent the chromatographic peaks of 14 compounds. The 14 compounds are Momordicoside O (1), Momordicoside E (2), Momordicoside S (3), Momordicoside A (4), Goyaglycoside h (5), Momorcharaside B (6), Momordicoside Q/Karaviloside XI (7), Momorcharaside M/N/Karaviloside Ⅹ (8), Goyasaponin I (9), Goyasaponin II (10), Goyalycoside e/f (11), Momordicoside L (12), Momordicoside P (13) and Momordicin II (14) respectively.).

The chemical components of MCE were analyzed using UHPLC-MS and MS/MS. The molecular mass was determined using full scanning mass spectrometry in positive and negative ion modes. The mass error was obtained by calculating the difference between the measured molecular mass and the theoretical value of the compound. The mass error was less than 10 ppm in this identification experiment. In the positive ion mode, the [M + H]^+^ and sodium adduct [M + Na]^+^ ions were mainly generated, while in the negative ion mode, the compounds showed deprotonated [M − H]^−^ ions and [M + HCOO]^−^ ions. A preliminary analysis of the results showed that the chromatographic peaks generated in the negative mode were more abundant and the MS/MS compound fragment information was much clearer, which was beneficial to identifying the compounds. Therefore, the negative mode was chosen for the following process of analysis. The total ion chromatogram (TIC) of MCE (Figure 1) showed that the chromatographic peaks were well separated, and all compounds were preliminarily characterized by analyzing the UHPLC-MS and MS/MS data and comparing them with the relevant literature. 

**Figure 2:**
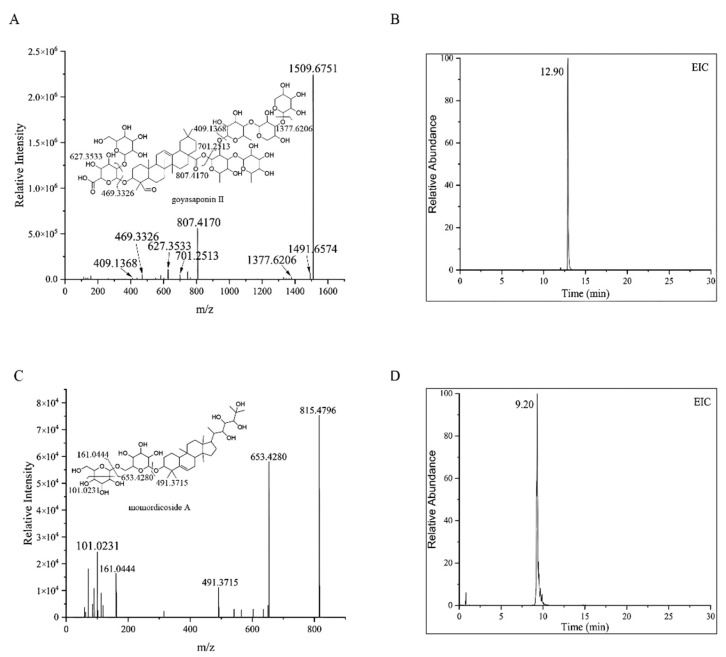
MS/MS spectrum (**A**) and the EIC (**B**) of goyasaponin II and MS/MS spectrum (**C**) and the EIC (**D**) of momordicoside A.

**Figure 3:**
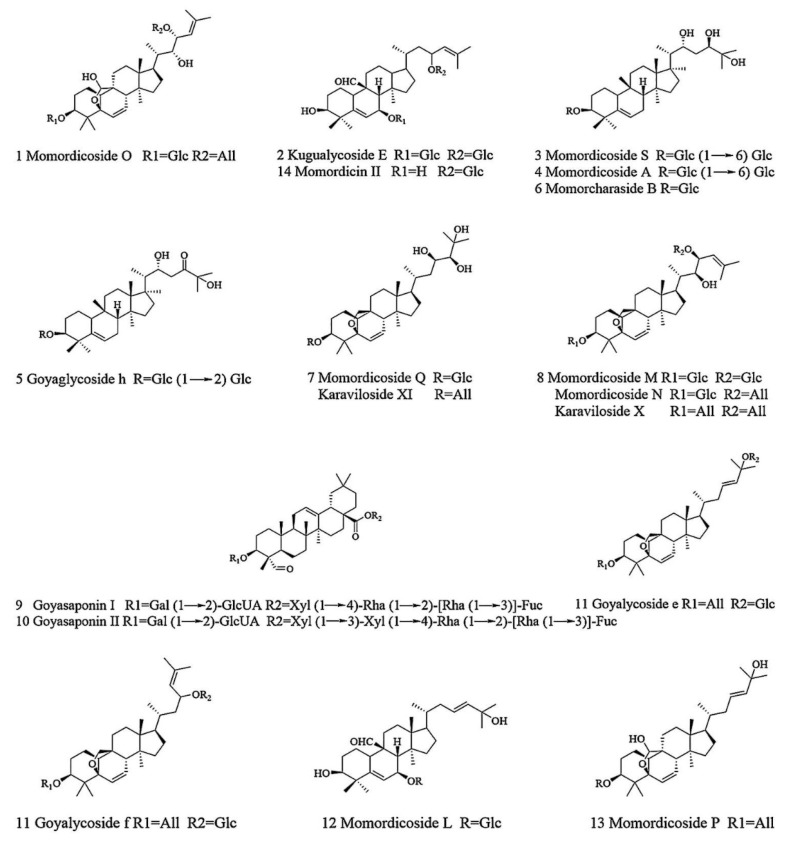
The structures of the compounds identified in MCE.

Finally, a total of 14 major compounds, triterpenoids, were identified in MCE. The *m*/*z* values of the ions are expressed as integers to facilitate the statement in discussion. The glycosides of these triterpenoids are normally composed of β-d-glucopyranosyl (β-d-Glc), β-d-xylopyranosyl (β-d-Xyl), β-d-galactopyranosyl (β-d-gal), α-L-rhamnopyranosyl (α-L-Rha), β-d-fucopyranosyl (β-d-Fuc) and β-d-allopyranosyl (β-d-All), or in the form of disaccharides or multiple saccharide chains, linked by O-bond substituents at different locations of aglycones. The characteristic neutral losses of these sugar residues are 132 Da (fructose), 146 Da (deoxyhexose) and 162 Da (hexose), respectively. The MS/MS spectra and MS extracted ion chromatogram (EIC) of goyasaponin II and momordicoside A are shown in Figure 2. These two representative compounds are taken as examples in the following discussions to explain the identification of the compounds. As shown in Figure 2A,B, the [M − H]^−^ ion at *m*/*z* 1509 of goyasaponin II produces a fragment ion at *m*/*z* 1377 by the loss of α-L-Xyl residue (132 Da), an *m*/*z* 807 ion is produced by the loss of Xyl (1→3) Xyl (1→4) Rha (1→2) [Rha (1→3)] Fuc residue (132 Da + 132 Da + 146 Da + 146 Da + 146 Da), and an *m*/*z* 469 ion is produced by the loss of Gal (1→2) glucuronic acid residue on the basis of an *m*/*z* 807 ion. Momordicoside A is a tetracyclic triterpene of cucurbitane type with the molecular formula C_42_H_72_O_15_ and a molecular weight of 816. The [M − H]^−^ ion of momordicoside A (*m*/*z* 815) at 9.20 min can be clearly observed in Figure 2C,D. The fragment ion at *m*/*z* 653 is produced by the loss of Glc residue (162 Da), and *m*/*z* 491 is generated by the loss of Glc (1→6) Glc (162 Da + 162 Da) residue. The information regarding the identification of the compounds is shown in Table 1, and the structures are shown in Figure 3.

### 2.2. Behavioral Assessments

#### 2.2.1. Effects of MCE on Morris Water Maze (MWM) Test

**Figure 4:**
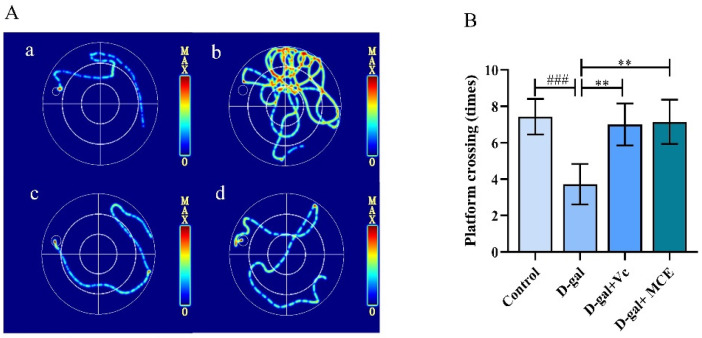
Heat maps of spatial navigation in MWM test (**A**): control group (**a**), d-gal group (**b**), d-gal + Vc group (**c**), d-gal + MCE group (**d**); and platform crossing (times) of spatial trial in MWM test (**B**). Data were reported as the mean ± S.D. of 20 mice in each group. ^###^ *p* < 0.001 vs. control group; ** *p* < 0.01 vs. d-gal group.

Cognitive impairment along with a decline in learning and memory are symptoms of brain dysfunction that are closely related to aging. In a spatial navigation test, it was clear that long-term d-gal treatment resulted in a longer escape latency (*p* < 0.001), indicating that the mice developed severe cognitive impairment and that the model was successfully established (Table 2). The mice treated with vitamin c (Vc) and MCE were able to find the platform in a short escape latency, which is in sharp contrast with the d-gal group (*p* < 0.001). Moreover, the heat maps of spatial navigation, shown in Figure 4A, provide information about the movement path of the mice, enhancing the observability and reliability of the experiment. The mice in the d-gal group had a chaotic swimming route and most of them could not find the platform, while the mice in the MCE group could find the platform within a short distance and time. In a probe trial, the mice in the d-gal group mostly moved in the quadrant without a platform and spent a remarkably shorter time in the target quadrant compared with the control group (*p* < 0.001). Further, the platform crossing times of the d-gal group were notably fewer than those of the MCE group (*p* < 0.01), illustrated in Figure 4B.

#### 2.2.2. Effects of MCE on Step-Down Test

After training, the escape latency and error times of the step-down test in each group were calculated, and the results are shown in Table 3. The d-gal group shows a shorter escape latency and higher error times compared with the control group. As for the MCE group, the escape latency is longer (*p* < 0.001) and the number of errors is significantly lower than that in the d-gal group (*p* < 0.01). The above again indicates that a long-term injection of d-gal can damage the learning and memory ability of mice, establishing the success of the model. 

### 2.3. Effects of MCE on Oxidant Status In Vivo

**Figure 5:**
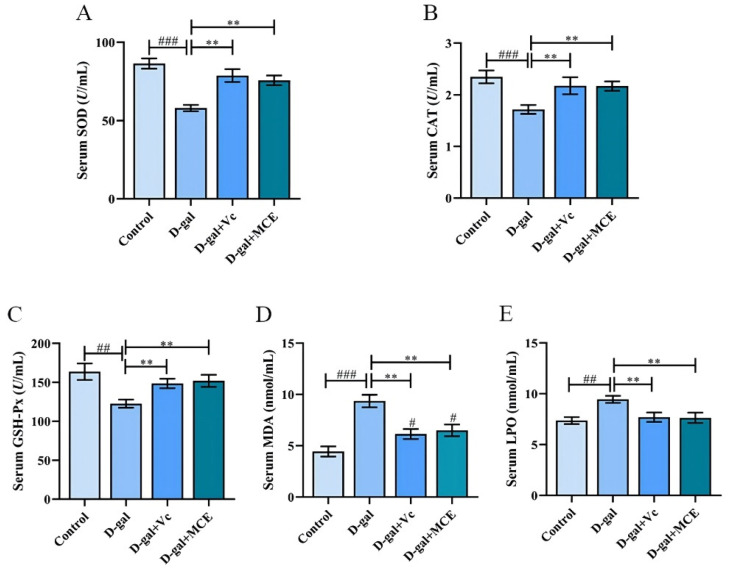
Effects of MCE on the activity of antioxidant enzymes and the level of lipid peroxidation. SOD (**A**), CAT (**B**), GSH-Px (**C**), MDA (**D**), LPO (**E**). Data were reported as mean ± S.D. of 20 mice in each group. ^###^ *p* < 0.001, ^##^ *p* < 0.01, ^#^ *p* < 0.05 vs. control group; ** *p* < 0.01 vs. d-gal group.

The determination results of antioxidant enzyme activity in vivo are shown in Figure 5. It can be seen that the activities of SOD, GSH-Px and CAT in the d-gal group decrease dramatically and are significantly heightened after MCE treatment (*p* < 0.01). As for the level of lipid peroxidation, malondiadehyde (MDA) and lipid peroxide (LPO) contents were measured. The MDA and LPO contents of the mice in the d-gal group show a significant increase and are both decreased by treatment with Vc and MCE with a significant downward trend (*p* < 0.01). The determination of these biochemical parameters indicates that long-term d-gal treatment resulted in severe oxidative damage in mice, while MCE can improve the oxidative stress of mice by increasing the activity of antioxidant enzymes to protect them from injury.

### 2.4. Effects of MCE on Body Weight and Organ Index

As shown in Table 4, the body weight of mice in the d-gal group decreases significantly compared with the control group (*p* < 0.01), and this weight loss can be reversed by MCE. In addition, the spleen and thymus indexes of the d-gal group are also significantly lower than those in the control group (*p* < 0.001, *p* < 0.01). There are no adverse effects on organ index after treatment with Vc and MCE. 

### 2.5. Effects of MCE on Histopathological Alterations

**Figure 6:**
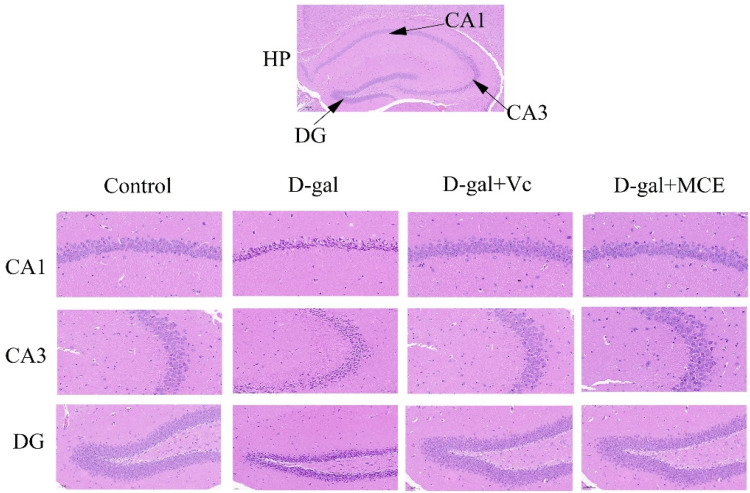
Effects of MCE on hippocampus (HP) with H&E staining (magnification 400×, scale bars 20 μm). (The cornu ammonis 1 (CA1), cornu ammonis 3 (CA3) and dentate gyrus (DG) regions of HP were selected for observation. In addition, Vc and MCE represent vitamin C and *Momordica charantia* L. ethanol extract respectively.)

The hippocampus (HP), located in the temporal lobe of the brain, is part of the limbic system and plays a crucial role in learning, memory and spatial positioning. Cornu ammonis 1 (CA1), cornu ammonis 3 (CA3) and dentate gyrus (DG) are important regions of the HP with complex structures and are influenced by a variety of factors. Therefore, these regions were selected for comparison and analysis in this experiment. H&E staining of the CA1, CA3 and DG regions have been highlighted in Figure 6. It can be seen that the cells in the CA1 and CA3 regions of the mice in the control group are plump with intact morphology and are arranged in an orderly fashion. There are approximately three or four layers of cells in the DG region with distinct layers, an exact structure, a uniform size and a tight arrangement. Moreover, the nuclei are round or oval and the chromatin is evenly distributed. In stark contrast, the cells in the CA1 region of the mice in the d-gal group are disordered, most of which change from round to angular or other irregular shapes, accompanied by karyopyknosis, deepened staining and an apparent decrease in neuronal cell density. A large number of cell deletions and vacuole-like structures can be observed in the CA3 region. Additionally, most of the cells in the DG region are degenerated and the nuclei is pyknotic. Although there are elements of cell loss and hyperchromatic phenomenon in the three regions of the Vc and MCE group, there are no obvious abnormalities compared with the control group, which indicates that the degree of pathological changes in the hippocampus can be mitigated by MCE treatment.

### 2.6. Effects of MCE on PI3K/AKT Signaling Pathway

**Figure 7:**
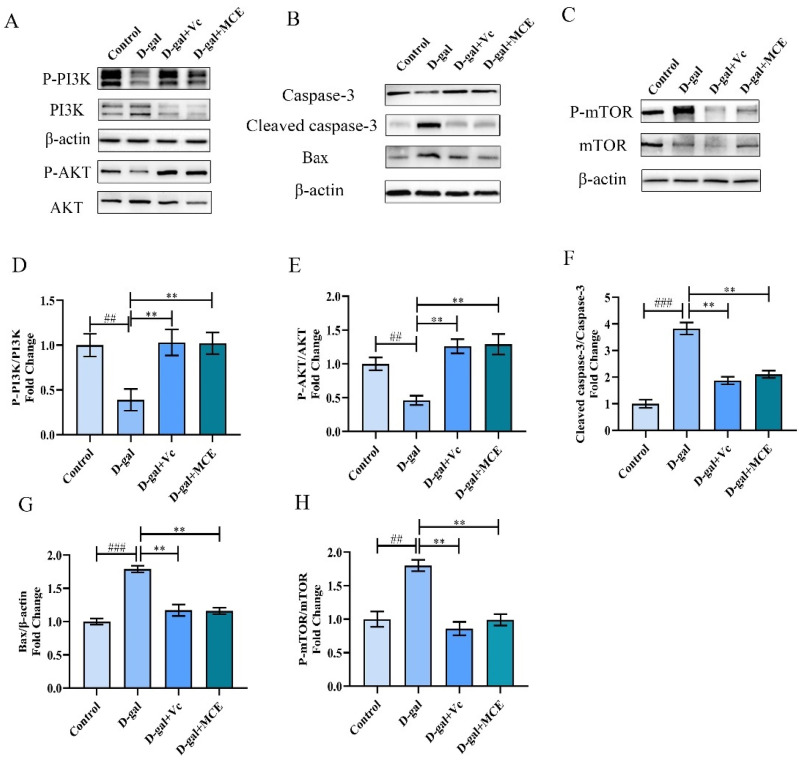
Effects of MCE on PI3K/AKT signaling pathway and downstream proteins. Representative Western blot images of P-PI3K, PI3K, P-AKT, AKT (**A**); caspase-3, cleaved caspase-3, Bax (**B**); P-mTOR, mTOR (**C**); histogram of P-PI3K/PI3K (**D**); P-AKT/AKT (**E**); cleaved caspase-3/caspase-3 (**F**); Bax/β-actin (**G**); P-mTOR/mTOR (**H**). All results were normalized by β-actin and expressed as a fold change relative to the gray value of the control group. ^###^ *p* < 0.001, ^##^ *p* < 0.01 vs. control group; ** *p* < 0.01 vs. d-gal group.

In order to reveal the anti-aging mechanism of MCE, the expression of aging-related proteins in the hippocampus of subacute aging mice after MCE intervention were detected in this study. As shown in Figure 7A,D,E, d-gal administration produced a significant reduction in the protein expression of P-PI3K/PI3K and P-AKT/AKT (*p* < 0.01). Simultaneously, a marked elevation in the expression levels of cleaved caspase-3/caspase-3 (*p* < 0.001), Bax/β-actin (*p* < 0.001) and P-mTOR/mTOR (*p* < 0.01) are illustrated in Figure 7B,C,F–H. However, the expression of these proteins in the MCE-treated mice shows opposite regulatory trends, which indicates that MCE could inhibit apoptosis and activate autophagy to avoid cell damage induced by d-gal in aging mice. 

## 3. Discussion

*Momordica charantia* L. is a common edible medicinal plant that contains various bioactive chemical components. Its chemical composition can be analyzed by nuclear magnetic resonance spectroscopy, but this requires high purity of the sample [26]. In this study, the UHPLC-Q-Exactive Orbitrap MS method was used for a qualitative analysis of its chemical components. UHPLC-MS has the advantages of being rapid with high resolution, high selectivity and sensitivity, making it easier to analyze the components of complex herbal mixtures by comparing their relative molecular mass, retention times, mass error and fragment ions [27]. By using the UHPLC-MS method, the compounds of MCE could be analyzed without separation or purification, which greatly shortened the experimental time and provided more data for the screening of monomeric compounds in the future.

Finally, 14 triterpenoids were detected and identified in MCE. The saponins were proven to have the ability to prolong the lifespan of elegans and yeast, as previously described in the introduction. As a result, it is reasonable to consider that triterpenoids are the main anti-aging substances in MCE. Of course, it cannot be denied that other unidentified substances may also have anti-aging activities, and this requires further research.

The free radical theory and the oxidative stress theory are widely accepted in research on the aging process, and previous studies have shown that an excessive accumulation of free radicals is the cause of many diseases, including aging [28]. Our results are consistent with these studies; a repeated injection of d-gal can weaken the activity of antioxidant enzymes and intensify lipid peroxidation in mice. Excessive free radicals can be eliminated by enhancing the activity of antioxidant enzymes or by supplementing exogenous antioxidants, so as to maintain the homeostasis of free radicals in the body and delay aging [29]. SOD, GSH-Px and CAT are all key enzymes in the antioxidant defense system. Therefore, SOD, GSH-Px and CAT activities are generally considered as markers of the aging process [30]. Our results showed that MCE could improve oxidative damage and recover the physiological functions in aging mice by enhancing the activity of these antioxidant enzymes. In addition, MDA is one of the oxidation end products produced by the peroxidation reaction between free radicals and lipids in organisms, and with strong cytotoxicity, it can cause the cross-linking polymerization of biological macromolecules, such as proteins and nucleic acids, which aggravates membrane damage. LPO has the ability to cause changes in cell membrane fluidity and permeability, ulteriorly affecting the normal structure and function of cells. It is a type of product generated by lipid peroxidation reactions between reactive oxygen species (ROS) and phospholipids, enzymes, nucleic acids or any other biological macromolecules. The contents of MDA and LPO are closely related to aging [31]. In our study, after MCE treatment, the level of MDA and LPO in aging mice were significantly reduced. Therefore, it is reasonable to consider that MCE can improve the disordered free radical metabolism and reduce the level of lipid peroxidation by promoting the activity of antioxidant enzymes to exert anti-aging effects on d-gal-induced subacute aging in mice.

Further, it has been demonstrated that mice with an intraperitoneal administration of d-gal exhibited significant memory loss, weight loss and an atrophy of the spleen and thymus indexes in our studies. The weight loss may be due to the reduction in usual exercise and food intake. Thymus is an important lymphatic organ in vivo and the site where T-lymphocytes differentiate, develop and mature. Meanwhile, the spleen, the largest peripheral immune organ, is the main site of specific immunity. Spleen and thymus indexes are indicators to evaluate the development of immune organs in the body [32]. Aging bodies are often accompanied by a decline in immune function [33]. According to our study, MCE significantly improves the memory and learning abilities of mice and slows their weight loss. Simultaneously, there was no obvious abnormality in the immune organ index after MCE intervention, indicating that MCE can prevent a weakened immune ability in aging mice.

In order to examine the effects of MCE on aging mice from different perspectives, pathological observations of the hippocampus were also carried out. Previous studies have shown that the number and density of neurons in the hippocampus are inversely correlated with age [34]. In the process of aging, neurons undergo varying degrees of apoptosis, leading to a decrease in memory capacity, which is consistent with our observations for the d-gal group. However, MCE can reduce the apoptosis of hippocampal neurons as well as maintain the normal morphology and function of the hippocampus.

Previous studies have shown that apoptosis contributes to the aging process induced by d-gal [35]. Apoptosis is a type of physiological cell death that is essential for the formation and maintenance of homeostasis in humans and animals [36]. PI3K/AKT is one of the main signal transduction pathways closely related to cell proliferation, survival and apoptosis, and is affected by growth factors, insulin and many other factors [37]. AKT, activated by PI3K, could promote cell growth and survival via the phosphorylation of multiple cytoplasmic proteins in aging [38]. Bax, caspase-3 and Bcl-2 located downstream of the PI3K/AKT signaling pathway can strictly regulate apoptosis [39]. Among them, Bax, belonging to the Bcl-2 family, is one of the most important proapoptotic proteins that can cause apoptosis by increasing the permeability of mitochondrial membranes to activate the mitochondria-mediated apoptosis pathway. Caspase-3, a member of the caspase family, is responsible for initiating the caspase cascade that results in apoptosis and the cleaved caspase-3 is the active form of it. Studies have shown that active Bax and caspase-3 trigger widespread injury and degeneration, which accelerates the aging process [40,41]. However, AKT has a negative regulatory effect on Bax and inhibits the activation of caspase cascade reaction. In our experiments, a significant inhibition of the PI3K/AKT signaling pathway and a high expression of Bax and cleaved caspase-3 were detected after d-gal treatment, while MCE could reverse this situation, MCE can directly or indirectly activate AKT by activating PI3K, with the activated AKT achieving a negative regulation of proapoptotic protein Bax and caspase 3, thus promoting cell proliferation, survival and a reduction in apoptosis. Therefore, we concluded that the anti-aging effect of MCE might be achieved by activating the PI3K/AKT signaling pathway and inhibiting the expression of Bax and caspase-3.

mTOR belongs to the PIKK family and is closely related to protein synthesis and autophagy in the body. mTOR promotes protein synthesis when it is highly expressed in an appropriate range but inhibits autophagy when over-regulated [42,43]. Apoptosis and autophagy, two important physiological processes with distinct differences and mutual influences, play an important role in the aging process. Autophagy is a natural regulatory mechanism that breaks down defective or old proteins into amino acids and reapplies them to build new proteins and tissues. This is a regenerative process that affects longevity and anti-aging, making cells healthier and more active. In recent years, many studies have focused on prolonging the lifespan of organisms by inhibiting the high expression of mTOR to activate autophagy [44,45]. Our data indicate that MCE could relieve the autophagy inhibition caused by long-term d-gal treatment by reducing the level of P-mTOR/mTOR, thereby promoting cell self-renewal in aging mice.

## 4. Materials and Methods

### 4.1. Chemicals and Reagents

Fresh *Momordica charantia* L. was obtained from Shouguang country (Shandong, China), identified by Dr. Bo Li (Changchun University of Chinese Medicine, Changchun, China) and stored in the Jilin Ginseng Academy with voucher specimen No. 20200016. Both the HPLC-grade acetonitrile (ACN) and formic acid were purchased from Fisher Scientific (Waltham, MA, USA). The Milli-Q^®^ IQ 7000 system (Darmstadt, Germany) was used to purify 18 MΩ water. d-gal (99% purify) and Vc were purchased from Sigma Chemical Co. (St. Louis, MO, USA). Ethanol and other chemicals were of analytical grade. Commercial kits for the measurement of CAT, SOD, GSH-Px, MDA and protein content (bicinchoninic acid, BCA) were obtained from Nanjing Jiancheng Biotech. Co., Ltd. (Nanjing, China). An ELISA kit for the determination of lipid peroxides (LPO) was purchased from Yi Feixue Biotech. Co., Ltd. (Nanjing, China). Polyvinylidene fluoride (PVDF) membranes (0.45 μm), RIPA, cocktail, PMSF, phosphorylase buffer, protein loading buffer and quick blocking buffer were obtained from Servicebio Co., Ltd. (Wuhan, China). PI3K, P-PI3K (Tyr458-199), AKT, P-AKT (Ser 473), caspase-3, cleaved caspase-3, Bax, mTOR, P-mTOR (Ser 2448), β-actin antibodies (rabbit anti-mouse) and horseradish peroxidase-conjugated antibody (goat anti-rabbit) were acquired from Affinity Biosciences Co., Ltd. (Jiangsu, China). ECL chemiluminescent substrate was obtained from New Cell & Molecular Biotech. Co., Ltd. (Jiangsu, China).

### 4.2. Preparation of MCE

In this experiment, the fruit part of the plant was selected. The pulp and seeds were removed after washing, then the fruit was cut into 1 cm thick slices and placed in an oven at 105 °C for 20 min first, then transferred to an oven at 45 °C for drying to obtain dried samples. Soon afterwards, the samples were ground into powder, then immersed in 70% aqueous ethanol (ethanol: water, 70:30, *v*/*v*) and extracted in an ultrasonic bath at 45 °C for 30 min. After repeating the extraction 3 times, the 3 aliquots were combined and centrifuged at 10,000 rpm for 10 min, then the MCE was prepared. Part of the MCE was filtered through a 0.45 μm filter for analysis of UHPLC-MS, while the other was evaporated under nitrogen and redissolved with water for intragastric administration in mice.

### 4.3. UHPLC-MS Analysis

UHPLC analysis was performed on an Ultimate 3000 high-performance liquid chromatography system equipped with a PDA detector and a hybrid quadrupole high-resolution accurate mass (HRAM) Orbitrap (Thermo Fisher Scientific, Sunnyvale, CA, USA) system. The instrument was calibrated to reduce errors by using a calibration solution. A reversed-phase BEH RP-18 column (2.1 mm × 100 mm, particle size 1.7 μm, Waters, Milford, MA, USA) balanced at 45 °C was used for chromatographic separation. Mobile phase A (0.1%, *v*/*v*, aqueous formic acid) and mobile phase B (100%, ACN) were used with the flow rate of 0.3 mL/min. The liner gradient elution was carried out as follows: 0–2 min (5–20% B), 2–8 min (20–40% B), 8–15 min (40–45% B), 15–25 min (45–60% B), 25–28 min (60–95% B) and 28–30 min (95–5% B). The injection volume was 3.0 µL, and samples were kept at the temperature of 4 °C.

MS spectrometric detection was performed on a Q-Exactive Orbitrap MS (Thermo Fisher Scientific, Sunnyvale, CA, USA) equipped with an electrospray ionization (ESI) source. The ESI source was configured as follows: the full scan and ddMS2 mode in both positive and negative ion modes were selected in this experiment over an *m*/*z* range of 200–2000. The sheath gasflow was 6.125 Mpa, the aux gasflow was 2.625 Mpa and the sweep gasflow was 0.175 Mpa. The capacity temperature was 300 °C and the capillary voltage was 3.5 kv. All data were exported by SIEVE (Version 2.1, Agilent Technologies, Santa Clara, CA, USA) and analyzed by Xcalibur 4.1 software (Thermo Fisher Scientific, Sunnyvale, CA, USA).

### 4.4. Animal Experiments

Eight-week-old male Kunming mice (weight: 20 ± 2 g) were obtained from Changsheng Laboratory Animal Technology Co., Ltd., Liaoning, China (permit number: SCXK2020-0001). The animal experiments were approved by the Institutional Animal Care and Use Committee (IACUC) of the Changchun University of Chinese Medicine (permit number: CPCCUCM IACUC 2021-062). After a 1-week acclimation to the laboratory environment (constant temperature of 23 ± 2 °C, 50–60% humidity, 12 h light/dark cycle and adequate food and water), the mice were divided into four groups randomly (*n* = 20): the control group (healthy mice with administration of water by gavage), the d-gal group (healthy mice with intraperitoneal injections of 1.35 g/kg d-gal and administration of water by gavage), the Vc group (d-gal mice treated with Vc by gavage at 100 mg/kg/d) and the MCE group (d-gal mice treated with MCE by gavage at 500 mg/kg/d); the volume of gavage in the control and d-gal groups was equivalent. The whole experiment lasted 6 weeks.

### 4.5. Behavioral Assessments

#### 4.5.1. MWM Test

In this study, the MWM test was used to evaluate the spatial learning and memory ability of d-gal-induced aging in mice. The equipment comprised a circular tank, a movable platform, an automatic video camera and a computer for recording and analysis. The experiment was performed according to previous descriptions [46,47]. The whole experiment was divided into two parts: spatial navigation and probe trial. Each group was trained for four consecutive days, and the escape latency and location heat maps of the mice were monitored by video on the fifth day. In each spatial navigation training session, the mice were released in a maze facing the pool wall from four different directions and allowed to swim freely for 60 s. Escape latency was the time taken by the mice to find the platform from entering the water. Guidance was needed when mice failed to reach the platform within 60 s. All mice were kept on the platform for 15 s to form memories, regardless of whether they found the platform. On the sixth day, non-toxic black dye was added before the start of the spatial probe trial and the platform was removed. Each mouse was allowed to swim freely within 60 s, with the target quadrant crossing times, the dwell time and the platform crossing times recorded.

#### 4.5.2. Step-Down Test

The step-down test was a passive avoidance experiment, also used to assess learning and memory ability, in which the mice were trained to jump onto an insulated platform to avoid the electric copper grid [48]. The apparatus consisted of a plurality of rectangular reflection boxes separated by plastic plates, with an electric copper grid at the bottom. Each individual box was equipped with an insulated platform 5 cm in diameter. The mice were trained before the test, they were placed in the box to familiarize themselves with the environment, then 36 V alternating current was applied for 5 min. The mice were placed on the platform 24 h after the training, and an image recorder connected to a computer was used to record the latency and error times.

### 4.6. Biological Sample Collection and Preparation

After the behavioral assessments, the mice in all groups were fasted for 24 h. Blood samples were obtained from the retrobulbar vein and preserved in sterile tubes, centrifuged at 3000 rpm for 10 min at 4 °C. Then, the supernatant serum was separated and snap-frozen with liquid nitrogen and finally stored separately at −80 °C. The determination of biochemical parameters (SOD, GSH-Px, CAT activities as well as the accumulation content of MDA and LPO) in the serum samples was carried out according to the manufacturer’s protocol. The spleens and thymuses were obtained for the calculation of organ index (organ weight/body weight (mg/g)) [49]. Simultaneously, the hippocampi were excised from the brains, part of which were fixed with paraformaldehyde for over 24 h before histological examination, and the rest were used for Western blot analysis.

### 4.7. Histopathological Examination

The fixed hippocampi were observed by H&E staining [50]. Briefly, tissues were dehydrated using different dilutions of ethanol, remaining briefly in xylene before the addition of paraffin. The paraffin blocks were precooled for more than 30 min, sliced into 4 μm sections and stained with hemotoxylin and eosin (H&E) for microscopic observation.

### 4.8. Western Blot Analysis

The appropriate hippocampi were weighed and immersed in a complete RIPA (RIPA:Cocktail:PMSF:Phosphorylase inhibitor = 100:2:1:1) buffer, then homogenized with a low-temperature grinding instrument, followed by an ice bath for 30 min to lyse completely. Later, centrifuged at 10,000 rpm for 10 min, the supernatant was collected for protein content determination using a BCA protein assay kit. Finally, a protein loading buffer was added, and the samples were boiled for 10 min. An amount of 10 μL of the sample (20 μg protein) was separated on 6–10% sodium dodecyl sulfate (SDS)-polyacrylamide gels (Servicebio, Wuhan, China), electrophoresed and transferred to PVDF membranes. The membranes were incubated with PI3K, P-PI3K, AKT, P-AKT, caspase-3, Bax, mTOR, P-mTOR and β-actin antibodies at 4 °C for 24 h after quick blocking. Then, a horseradish peroxidase-conjugated secondary antibody was added to incubate for 1 h at an ambient temperature with shaking. Finally, the membranes were developed with an ECL kit, visualized and captured by an imaging system (Tanon, Shanghai, China). Image J software was used to analyze the gray value of the target proteins.

### 4.9. Statistical Analysis

All data were presented as mean ± S.D. and analyzed using GraphPad PRISM version 8.0.2 software (GraphPad Software, San Diego, CA, USA). The differences between the two groups were determined using Student’s *t*-test, while a one-way analysis of variance (ANOVA), followed by Tukey’s post hoc test, was used to compare multiple groups. It was considered statistically significant when *p* < 0.05.

## 5. Conclusions

This experiment was the first to demonstrate the anti-aging effect of *Momordica charantia* L. on subacute aging mice. The study of the chemical components of *Momordica charantia* L. indicated that the anti-aging effect may be closely related to triterpenoids. The biochemical and pharmacological parameters indicated that *Momordica charantia* L. could maintain redox balance and reduce oxidative stress in aging mice. It has the potential to promote cell survival and to reverse the decline in learning and memory ability caused by d-gal-induced subacute aging in mice by activating the PI3K/AKT signaling pathway. Furthermore, protective autophagy was activated by inhibiting the overactivation of mTOR. As a result, *Momordica charantia* L. may be an efficient treatment in aging, which warrants further research.

## Figures and Tables

**Table 1:** Compounds identified in MCE using UHPLC-MS and MS/MS.

Peak	t_R_ min	Measured *m*/*z*	Mass Error (Δppm)	Molecular Formula	Adduct	MS/MS	Compound
1	7.43	857.4567	3.1	C_42_H_68_O_15_	[M + HCOO]^−^	179.0548, 631.3800 161.0444, 101.0230	Momordicoside O
2	8.17	843.4779	3.7	C_42_H_70_O_14_	[M + HCOO]^−^	797.4688, 635.4132 617.4055, 161.0444	Kugualycoside E
3	8.18	977.5341	1.4	C_48_H_82_O_20_	[M − H]^−^	815.4693, 797.4680 161.0444, 179.0550	Momordicoside S
4	9.20	815.4794	−0.5	C_42_H_72_O_15_	[M − H]^−^	653.4269, 491.3715 161.0444	Momordicoside A
5	9.65	859.4731	4.0	C_42_H_70_O_15_	[M + HCOO]^−^	813.4646, 651.4112 633.3995, 489.3572	Goyaglycoside h
6	11.26	699.4344	2.7	C_36_H_62_O_10_	[M + HCOO]^−^	653.4269, 491.3735 161.0444	Momorcharaside B
7	11.40	697.4191	3.2	C_36_H_60_O_10_	[M + HCOO]^−^	651.4116, 633.3973 489.3567, 161.0442	Momordicoside Q/ Karaviloside XI
8	12.00	841.4617	3.1	C_42_H_68_O_14_	[M + HCOO]^−^	795.4536, 633,4033 615.3884, 161.0442	Momorcharaside M/N Karaviloside Ⅹ
9	12.58	1377.6312	−1.5	C_65_H_102_O_31_	[M − H]^−^	1377.6312, 807.4169 627.3515, 469.3347	Goyasaponin I
10	12.90	1509.6751	−0.3	C_70_H_110_O_35_	[M − H]^−^	1509.6751, 627.3533 807.4170, 409.1368	Goyasaponin II
11	13.02	825.4681	4.7	C_42_H_68_O_13_	[M + HCOO]^−^	779.4583, 617.4055 161.0443, 101.0230	Goyalycoside e/f
12	15.38	679.4094	4.6	C_36_H_58_O_9_	[M + HCOO]^−^	633.4007, 471.3479 161.0443, 101.0230	Momordicoside L
13	17.28	679.4091	4.1	C_36_H_58_O_9_	[M + HCOO]^−^	633.4006, 471.3443 161.0443, 101.0230	Momord icoside P
14	17.87	679.4088	3.7	C_36_H_58_O_9_	[M + HCOO]^−^	633.4030, 455.3177 161.0444	Momordicin II

**Table 2:** Effects of MCE on escape latency, target quadrant crossing times and dwell time in MWM test.

Groups	Escape Latency (s)	Target Quadrant Crossing Times	Dwell Time (s)
Control	10.39 ± 1.91	12.00 ± 0.82	49.05 ± 6.86
d-gal	45.17 ± 5.13 ^###^	5.00 ± 0.82 ^###^	19.50 ± 3.31 ^###^
d-gal+ Vc	12.5 ± 1.65 ***	10.50± 1.29 **	40.4 ± 4.32 **
d-gal+ MCE	13.07 ± 2.57 ***	11.75 ± 0.96 ***	39.13 ± 5.47 **

Data were reported as the mean ± S.D. of 20 mice in each group. ^###^ *p* < 0.001 vs. control group; *** *p* < 0.001, ** *p* < 0.01 vs. d-gal group.

**Table 3:** Effects of MCE on escape latency and error times of step-down test.

Groups	Escape Latency (s)	Error Times	Dwell Time (s)
Control	277.89 ± 19.77	1.17 ± 0.75	49.05 ± 6.86
d-gal	84.56 ± 20.24 ^###^	3.33 ± 1.03 ^##^	19.50 ± 3.31 ^###^
d-gal+ Vc	262.63 ± 32.58 ***	1.00 ± 063 **	40.4 ± 4.32 **
d-gal+ MCE	262.15 ± 26.41 ***	1.17 ± 0.75 **	39.13 ± 5.47 **

Data were reported as the mean ± S.D. of 20 mice in each group. ^###^ *p* < 0.001, ^##^ *p* < 0.01 vs. control group; *** *p* < 0.001, ** *p* < 0.01 vs. d-gal group.

**Table 4:** Effects of MCE on body weight and organ index.

Groups	Body Weight (g)	Organ Index	
		Spleen (mg/g)	Thymus (mg/g)
Control	47.86 ± 3.15	3.65 ± 0.04	1.50 ± 0.07
d-gal	41.25 ± 2.46 ^##^	2.00 ± 0.08 ^###^	0.84 ± 0.17 ^##^
d-gal + Vc	45.68 ± 2.32 **	3.43 ± 0.15 **	1.42 ± 0.10 **
d-gal + FS	45.88 ± 2.65 **	3.49 ± 0.11 ***	1.45 ± 0.04 **

Data were reported as mean ± S.D. of 20 mice in each group. ^###^ *p* < 0.001, ^##^ *p* < 0.01 vs. control group; *** *p* < 0.001, ** *p* < 0.01 vs. d-gal group.

## Data Availability

All the data used in this study are available within this article. Further inquiries can be directed to the authors.

## Sample Availability

Samples of the compounds are not available from the authors.

## References

[1] López-Otín C., Blasco M.A., Partridge L., Serrano M., Kroemer G. (2013). The hallmarks of aging. Cell.

[2] Garg G., Singh S., Singh A.K., Rizvi S.I. (2017). Antiaging Effect of Metformin on Brain in Naturally Aged and Accelerated Senescence Model of Rat. Rejuvenation Res..

[3] Zhang Y., Zhang J.J., Wang S.X. (2021). The Role of Rapamycin in Healthspan Extension via the Delay of Organ Aging. Ageing Res. Rev..

[4] Perls T. (2013). The Reappearance of Procaine Hydrochloride (Gerovital H3) for Antiaging. J. Am. Geriatr. Soc..

[5] Shen C.Y., Jiang J.G., Li Y., Wang D.W., Zhu W. (2017). Anti-ageing active ingredients from herbs and nutraceuticals used in traditional Chinese medicine: Pharmacological mechanisms and implications for drug discovery. Br. J. Pharmacol..

[6] Wang S.Z., Li Z.L., Yang G.L., Ho C.T., Li S.M. (2017). *Momordica charantia*: A popular health-promoting vegetable with multifunctionality. Food Funct..

[7] Oliveira M.S., Almeida W., Wariss F., Bezerra F. (2018). Phytochemical profile and biological activities of *Momordica Charantia* L. (Cucurbitaceae): A review. Afr. J. Biotechnol..

[8] Habtemariam S. (2019). The Chemical and Pharmacological Basis of Bitter Melon (Momordica charantia L.) as a Potential Therapy for Type 2 Diabetes and Obesity—Medicinal Foods as Potential Therapies for Type-2 Diabetes and Associated Disease.

[9] Grover J.K., Yadav S.P. (2004). Pharmacological actions and potential uses of *Momordica charantia*: A review. J. Ethnopharmacol..

[10] AkyÜz E., TÜrkoĞlu S., BaŞkan K.S., TÜtemet E., Apak M.R. (2020). Comparison of antioxidant capacities and antioxidant components of commercial bitter melon (*Momordica charantia* L.) products. Turk. J. Chem..

[11] Villarreal-La Torre V.E., Guarniz W.S., Silva-Correa C., Cruzado-Razco L., Siche R. (2020). Antimicrobial activity and chemical composition of *Momordica charantia*: A review. Pharmacogn. J..

[12] Patel R., Mahobia N., Upwar N., Waseem N., Talaviya H., Patel Z. (2010). Analgesic and antipyretic activities of *Momordica charantia* Linn. fruits. J. Adv. Pharm. Technol..

[13] Dandawate P.R., Subramaniam D., Padhye S.B., Anant S. (2016). Bitter melon: A panacea for inflammation and cancer. Chin. J. Nat. Med..

[14] Angamuthu D., Purushothaman I., Kothandan S., Swaminathan R. (2019). Antiviral study on *Punica granatum* L. *Momordica charantia* L. *Andrographis paniculata* Nees, and *Melia azedarach* L. to Human Herpes Virus-3. Eur. J. Integr. Med..

[15] Kim K.B., Lee S., Heo J.H., Kim J.H. (2017). Neuroprotective effects of *Momordica charantia* extract against hydrogen peroxide-induced cytotoxicity in human neuroblastoma SK-N-MC cells. J. Nutr. Health.

[16] Dennam H. (1956). Aging: A theory based on free radical and radiation chemistry. J. Gerontol..

[17] Bjorksten J. (1968). The crosslinkage theory of aging. J. Am. Geriatr. Soc..

[18] Bauer M.E., Fuente M.D.L. (2014). Oxidative Stress, Inflammaging, and Immunosenescence—Inflammation, Advancing Age and Nutrition.

[19] Cefalu C.A. (2011). Theories and mechanisms of aging. Clin. Geriatr. Med..

[20] Lin C.X., Chen Y., Lin Y.Z., Wang X.B., Hu L.Y., Cao Y., Chen Y.J. (2020). Antistress and anti-aging activities of *Caenorhabditis elegans* were enhanced by *Momordica saponin* extract. Eur. J. Nutr..

[21] Cao X.L., Sun Y.J., Lin Y.F., Pan Y.J., Farooq U., Xiang L., Qi J.H. (2018). Antiaging of Cucurbitane Glycosides from Fruits of *Momordica charantia* L.. Oxid. Med. Cell. Longev..

[22] Schieber M., Chandel N.S. (2014). ROS function in redox signaling and oxidative stress. Curr. Biol..

[23] Kong S.Z., Li J.C., Li S.D., Liao M.N., Li C.P., Zheng P.J., Guo M.H., Tan W.X., Zheng Z.H., Hu Z. (2018). Anti-Aging Effect of Chitosan Oligosaccharide on d-Galactose-Induced Subacute Aging in Mice. Mar. Drugs.

[24] Green D.R., Galluzzi L., Kroemer G. (2011). Mitochondria and the autophagy-inflammation-cell death axis in organismal aging. Science.

[25] Lapierre L.R., Kumsta C., Sandri M., Ballabio A., Hansen M. (2015). Transcriptional and epigenetic regulation of autophagy in aging. Autophagy.

[26] Yen P.H., Dung D.T., Nhiem N.X., Anh H.L.T., Hang D.T.T., Yen D.T.H., Cuc N.T., Ban N.K., Minh C.V., Kiem P.V. (2014). Cucurbitane-type triterpene glycosides from the fruits of *Momordica charantia*. Nat. Prod. Commun..

[27] Perez J.L., Jayaprakasha G.K., Patil B.S. (2019). Metabolite profiling and in vitro biological activities of two commercial bitter melon (*Momordica charantia* Linn.) cultivars. Food Chem..

[28] Kawagishi H., Finkel T. (2014). Unraveling the truth about antioxidants: ROS and disease: Finding the right balance. Nat. Med..

[29] Govindan S., Johnson E.E.R., Christopher J., Shanmugam J., Thirumalairaj V. (2016). Antioxidant and anti-aging activities of polysaccharides from *Calocybe indica* var. APK2. Exp. Toxicol. Pathol..

[30] Liguori I., Russo G., Curcio F., Bulli G., Aran L., Della-Morte D., Gargiulo G., Testa G., Cacciatore F., Bonaduce D. (2018). Oxidative stress, aging, and diseases. Clin. Interv. Aging..

[31] Moldogazieva N.T., Mokhosoev I.M., Mel’nikova T.I., Porozov Y.B., Terentiev A.A. (2019). Oxidative Stress and Advanced Lipoxidation and Glycation End Products (ALEs and AGEs) in Aging and Age-Related Diseases. Oxid. Med. Cell. Longev..

[32] Peng M.F., Fang Y.S., Miao M.S., Wang T. (2020). Effects of Wuweizi Syrup on Brain Aging Mice Model Induced by d-galactose. J. King Saud Univ. Sci..

[33] Akha A.A.S. (2018). Aging and the immune system: An overview. J. Immunol. Methods.

[34] Ji Z.H., Xu Z.Q., Zhao H., Yu X.Y. (2017). Neuroprotective effect and mechanism of daucosterol palmitate in ameliorating learning and memory impairment in a rat model of Alzheimer’s disease. Steroids.

[35] Yu Y., Bai F., Liu Y., Yang Y., Yuan Q., Zou D., Qu S., Tian G., Song L., Zhang T. (2015). Fibroblast growth factor (FGF21) protects mouse liver against d-galactose-induced oxidative stress and apoptosis via activating Nrf2 and PI3K/Akt path-ways. Mol. Cell. Biochem..

[36] Green D.R. (2022). The Mitochondrial Pathway of Apoptosis Part II: The BCL-2 Protein Family. Csh. Perspect. Biol..

[37] Ersahin T., Tuncbag N., Cetin-Atalay R. (2015). The PI3K/AKT/mTOR interactive pathway. Mol. Biosyst..

[38] Huang C.Y. (2020). Swimming exercise stimulates IGF1/PI3K/Akt and AMPK/SIRT1/PGC1α survival signaling to suppress apoptosis and inflammation in aging hippocampus. Aging.

[39] Caglayan C., Kandemir F.M., Darendelioğlu E., Küçükler S., Ayna A. (2021). Hesperidin protects liver and kidney against sodium fluoride-induced toxicity through anti-apoptotic and anti-autophagic mechanisms. Life Sci..

[40] Phu H.T., Thuan D.T.B., Nguyen T.H.D., Posadino A.M., Eid A.H., Pintus G. (2020). Herbal Medicine for Slowing Aging and Aging-associated Conditions: Efficacy, Mechanisms and Safety. Curr. Vasc. Pharmacol..

[41] Tong Q., Zhang M., Cao X., Xu S., Wang D., Zhao Y. (2017). Expression and activation of Daphnia pulex Caspase-3 are involved in regulation of aging. Gene.

[42] Liu G.Y., Sabatini D.M. (2020). mTOR at the nexus of nutrition, growth, ageing and disease. Nat. Rev. Mol. Cell. Biol..

[43] Weichhart T. (2018). mTOR as Regulator of Lifespan, Aging, and Cellular Senescence: A Mini-Review. Gerontology.

[44] Hurez V., Dao V., Liu A., Pandeswara S., Gelfond J., Sun L., Bergman M., Orihuela C.J., Galvan V., Padrón Á. (2016). Chronic mTOR inhibition in mice with rapamycin alters T, B, myeloid, and innate lymphoid cells and gut flora and prolongs life of immune-deficient mice. Aging Cell.

[45] Chang K., Kang P., Liu Y., Huang K., Miao T., Sagona A.P., Nezis I.P., Bodmer R., Ocorr K., Bai H. (2019). TGFB-INHB/activin signaling regulates age-dependent autophagy and cardiac health through inhibition of MTORC2. Autophagy.

[46] Chen P., Chen F., Lei J., Li Q., Zhou B. (2019). Activation of the miR-34a-Mediated SIRT1/mTOR Signaling Pathway by Urolithin A Attenuates d-Galactose-Induced Brain Aging in Mice. Neurotherapeutics.

[47] Jiang R., Gao J., Shen J., Zhu X., Wang H., Feng S., Huang C., Shen H., Liu H. (2020). Glycyrrhizic Acid Improves Cognitive Levels of Aging Mice by Regulating T/B Cell Proliferation. Front. Aging Neurosci..

[48] Zhang H., Wang Z., Li Y., Han J., Cui C., Lu C., Zhou J., Cheong L., Li Y., Sun T. (2018). Sex-Based Differences in Gut Microbiota Composition in Response to Tuna Oil and Algae Oil Supplementation in a d-Galactose-Induced Aging Mouse Model. Front. Aging Neurosci..

[49] Fu C.X., Dai L., Yuan X.Y., Xu Y.J. (2021). Effects of Fish Oil Combined with Selenium and Zinc on Learning and Memory Impairment in Aging Mice and Amyloid Precursor Protein Processing. Biol. Trace Elem. Res..

[50] Gong P., Wang D., Cui D., Yang Q., Wang P., Yang W., Chen F. (2021). Anti-aging function and molecular mechanism of Radix Astragali and Radix Astragali preparata via network pharmacology and PI3K/Akt signaling pathway. Phytomedicine.

